# Refining vitrectomy for proliferative diabetic retinopathy

**DOI:** 10.1007/s00417-023-06134-w

**Published:** 2023-06-14

**Authors:** San-Ni Chen, Shih-Jen Chen, Tsung-Tien Wu, Wei-Chi Wu, Chang-Hao Yang, Chung-May Yang

**Affiliations:** 1 Department of Ophthalmology, China Medical University Hospital, Taichung, Taiwan; 2 Department of Medicine, School of Medicine, China Medical University, Taichung, Taiwan; 3 Department of Ophthalmology, Taipei Veterans General Hospital, Taipei, Taiwan; 4 School of Medicine, National Yang Ming Chiao Tung University, Taipei, Taiwan; 5 Department of Ophthalmology, Kaohsiung Veterans General Hospital, Kaohsiung, Taiwan; 6 Department of Ophthalmology, Chang Gung Memorial Hospital, Linkou Medical Center, Taoyuan, Taiwan; 7 College of Medicine, Chang Gung University, Taoyuan, Taiwan; 8 Department of Ophthalmology, National Taiwan University Hospital, No. 7, Chung-Shan South Road, Taipei, 10002 Taiwan; 9 School of Medicine, National Taiwan University, Taipei, Taiwan

**Keywords:** Diabetic retinopathy, Fibrovascular proliferation, Pars plana vitrectomy, Retinal detachment, Vitreous hemorrhage

## Abstract

Pars plana vitrectomy (PPV) is the main treatment modality for patients with severe diabetic retinopathy. With the development of systems for microincision, wide-angle viewing, digitally assisted visualization, and intraoperative optical coherence tomography, contemporary PPV for diabetic retinopathy has been performed on a wider range of indications than previously considered. In this article, we reviewed, in conjunction with our collective experiences with Asian patients, the applications of new technologies for PPV in eyes with diabetic retinopathy and highlighted several important procedures and entities not generally reiterated in the literature, in order for vitreoretinal surgeons to optimize their approaches when facing the challenges imposed by the complications in diabetic eyes.

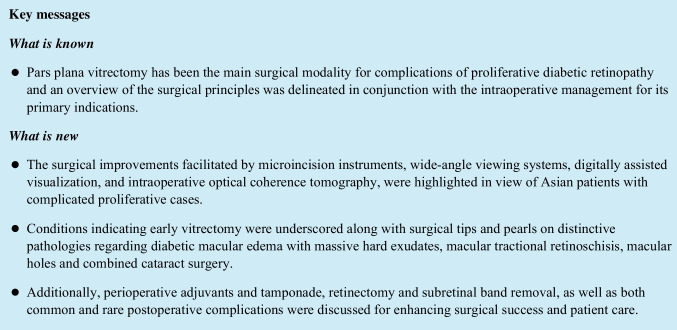

## Introduction

Pars plana vitrectomy (PPV) is the principle surgical technique to treat complications of diabetic retinopathy, namely, proliferative diabetic retinopathy (PDR), after non-surgical options such as panretinal laser photocoagulation (PRP) and intravitreal anti-vascular endothelial growth factor (anti-VEGF) injection are explored [1–3]. While fundamental principles remain, the latest developments in preoperative adjuncts, microincision instruments, and imaging systems propelled more versatile surgical techniques for a wider spectrum of diabetic retinopathy. The present article reviews recent advancements regarding vitrectomy for PDR, as reflected in the literature and through our consensus meetings. Importantly, our empirical experiences, while carrying practical values, should bring calls for further investigation. It is in this spirit that applications of new instruments, expanding surgical indications, and important surgical considerations are highlighted to delineate the new outlook of vitrectomy, as it becomes more refined for PDR.

## New instruments and technologies

### Microincision vitrectomy surgery (MIVS)

MIVS using 23- and 25-gauge (25G) instruments has been widely adopted to treat PDR. The latest addition of the 27-gauge (27G) system, while reducing wound leakage, has further achieved easier maneuverability within tight adhesion and narrower spaces, primarily aided by a beveled design and ultra-high cutter speed to tackle the complex fibrovascular proliferative tissue [4–6]. Our recent study comparing 25G and 27G systems showed a significant reduction to 66.3% in the use of forceps, which increased versatility and easy adaptation of the 27G system [7]. Other investigators also reported a significant reduction in perioperative complications including vitreous hemorrhage (VH), iatrogenic retinal breaks, and recurrent detachment [8, 9].

**Fig. 1:**
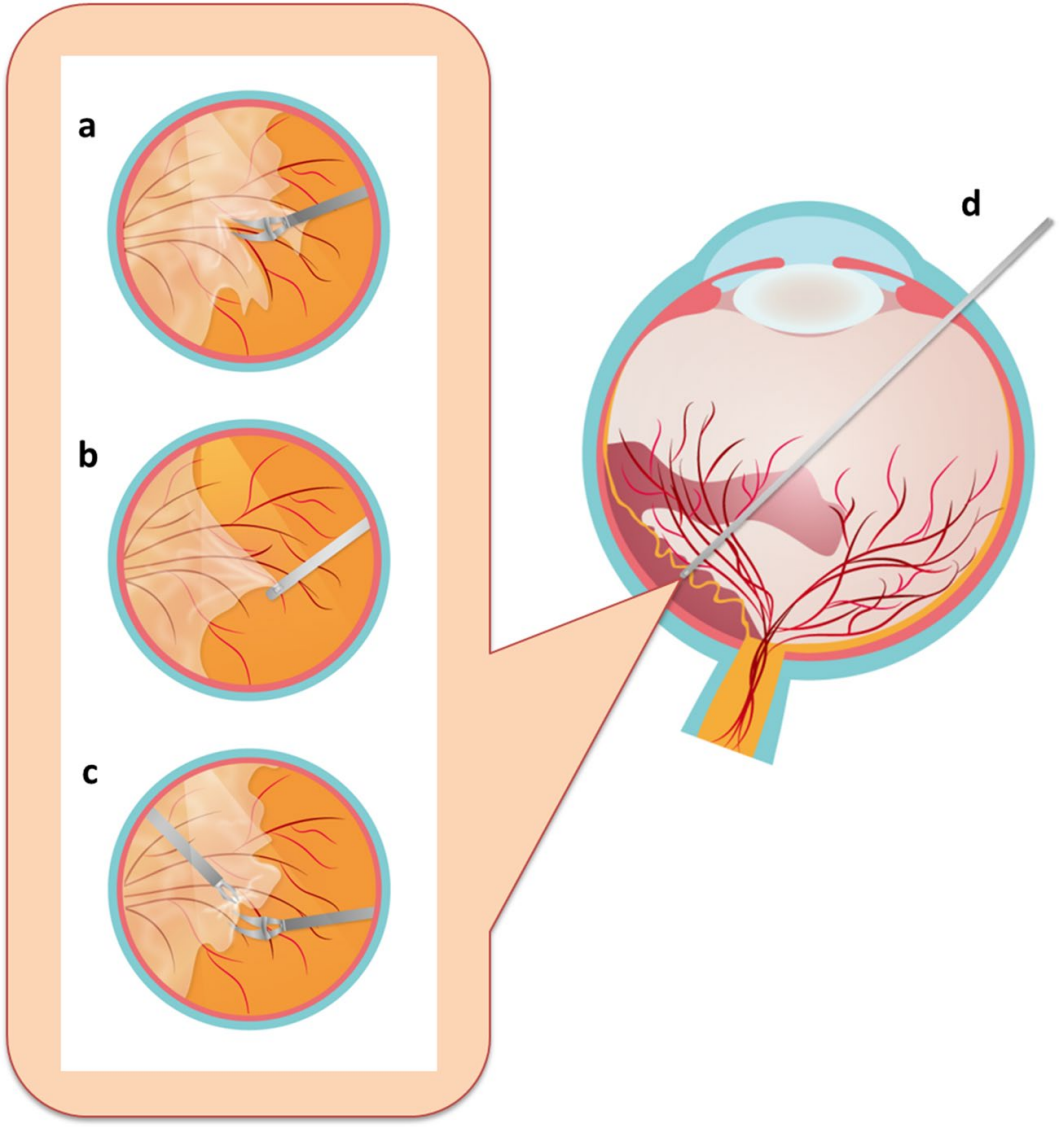
A schematic presentation of assorted vitrectors. Conventionally, different instruments are required to serve different functions. **a** illustrates conventional scissors for segmentation and delamination, **b** indicates a cutter to remove the vitreous, and **c** shows the bimanual incorporation of forceps to lift the membrane and scissors to cut. Nowadays, a small gauge vitrector as shown in **d** collectively serves the aforementioned purposes. The surgeon is able to cut and aspirate the vitreous, dissect the overlying membrane, and engage tissues by suction all in the same device

A fundamental advantage of the new 27G system is the substantial reduction of instrument changes and bimanual manipulation, as the beveled-tip vitrector can combine the functions of forceps, scissors, pick, and dissector all in one instrument (Fig. 1). The lift and shave technique described by Marie Berrocal [10] is conducive to the successful management of very adherent abnormal tissues, without inadvertent damages to the underlying retina. LED-illuminated instruments also improve the endoillumination of the 27G system [5, 6]. Additionally, we found that using a cutter speed of just 7500 cuts per minute (cpm), in spite of a maximum up to 20,000 cpm, is feasible for the efficient removal of very rigid proliferative membranes. This observation warrants further investigation. Despite these advantages, a larger gauge vitrector may still be required for the dissection of very thick membranes or at the far periphery, to facilitate global stabilization or eyeball rotation.

### Wide-angle viewing systems (WAVS)

Both contact and non-contact WAVS have been widely used for diabetic vitrectomy with comparable anatomical and functional outcomes [11]. While non-contact WAVS is especially convenient for Asian eyes with relatively small lid fissures and offers quality stereopsis for tissue delamination when using a 60D or 128D lens, the contact WAVS offers a wider view of the peripheral retina, up to 155° field of view by manufacturer, thus conferring easier access to the periphery as a surgeon rotates the eyeball. Furthermore, a specially designed equatorial contact lens with mid-range magnification allows concomitant stereopsis and clarity for peeling complex fibrovascular structures. The choice of the WAVS should always be finely balanced between factors such as pupil dilation, cataract severity, air/fluid filling, the field of view, and the resolution of imaging contrast, in conjunction with cost and operation logistics.

### Digitally assisted visualization system (DAVS)

As a heads-up viewing system, DAVS utilizes digital algorithms on a three-dimensional (3D) display to enhance various visual configurations, including light exposure, resolution, magnification, and depth of field. Using a smaller aperture, it reduces the need for endoillumination and compensates the compromised illumination in the conventional bimanual setup without causing phototoxicity [12, 13]. In our experiences, the 3D display also eliminates the need for frequent focus readjustment during membrane delamination at uneven retinal surfaces, which concurs with other surgeons favoring DAVS for macular surgeries. Although DAVS has been popular for good ergonomic comfort and educational value, it remains for individual surgeons to optimize their system of choice, since visualization systems with or without digital assistance are both adequate for diabetic vitrectomy.

### Intraoperative optical coherence tomography (iOCT)

The real-time information provided by microscope-integrated iOCT further refines vitrectomy for PDR, as the high-resolution imaging allows surgeons to promptly assess the retina initially obscured by dense VH or the complicated underlying fibrovascular structures, which are difficult to discern preoperatively. While better for aiding in the identification of various structural changes such as posterior vitreous detachment (PVD), vitreoschisis, pre-retinal membranes, and macular holes (MH), visualization of the surgical maneuvers between instruments and engaging membranes is also enhanced, thus facilitating intraoperative judgments and optimizing surgical performances in severe cases [14, 15]. However, some constraints are noted in the current iOCT systems, which include a small field of view, restricted depth of field, and potential distortion due to instrumental and maneuvering artifacts [14, 15].

## Surgical indications

The primary goals for vitrectomy are to remove non-clearing VH and release vitreoretinal traction, thus securing retinal attachment. Major surgical indications for diabetic vitrectomy are summarized as follows [3, 6, 16, 17].

### Non-clearing VH

Generally performed within 3–4 months of non-improvement, early vitrectomy is nevertheless recommended for patients with dense VH which obscures fundus examination or with B-scan ultrasonography showing retinal detachment (RD) threatening or involving the macula. Using MIVS and WAVS, functional improvement could reach 87% or higher, significantly better than the results reported in the early Diabetic Retinopathy Vitrectomy Study series [3, 16]. Additionally, the presence of rubeosis iridis is an important indication for prompt vitrectomy.

### Tractional retinal detachment (TRD)

Contraction of fibrovascular tissue at the vitreoretinal interface, often with subsequent retinal detachment, is a major indication for vitrectomy, particularly when the traction starts threatening the fovea or inducing tractional maculopathy. Among young diabetic patients below 40 years of age, an incidence of 76.5% was reported [18]. Early operation may also be necessary if traction exacerbates following PRP and/or anti-VEGF treatments [1–3].

### Combined tractional and rhegmatogenous retinal detachment (CTRRD)

Retinal breaks are likely to appear when tractional force acts on the thinning retina in PDR patients and are usually situated adjacent to the proliferative tissue or at previous laser scars. This condition warrants prompt surgery as RD may deteriorate rapidly.

### Progressive fibrovascular proliferation

The surgical removal of the fibrovascular tissue is essential to prevent deteriorating progression of fibrovascular proliferation (FVP). In severe cases known as anterior hyaloidal fibrovascular proliferation (AHFVP), FVP may grow in the peripheral retina, along the vitreous base and the anterior hyaloid surface, even after PRP or cryotherapy [19]. Vitrectomy for progressive FVP with adequate PRP prevents further traction which could induce maculopathies and severe anterior proliferation.

### Diabetic macular edema (DME)

Patients not responding to anti-VEGF after 3–5 monthly injections need to consider vitrectomy [20]. This is particularly important when DME is diffuse and resistant to adjunct laser treatment or is present with epiretinal membrane (ERM) or vitreomacular traction (VMT). ERM and inner limiting membrane (ILM) peeling, improving visual acuity in up to 90% of cases, may be employed to improve macular thickening, although the efficacy on visual acuity has not been studied beyond a 12-month follow-up [21, 22].

### Macular tractional retinoschisis (TRS)

As a frequent pattern of tractional macular elevation in PDR, macular TRS is characterized by the presence of bridging columnar tissues between the inner and outer layers [23]. The retina frequently shows more of the fibrous component than active and moderate proliferation. Vitrectomy with careful membrane removal usually achieves good anatomical outcomes, with complete resolution approaching 60% of the study cases.

### Macular holes and other vitreomacular interface abnormalities

MH in PDR patients may exist alone or be associated with RD, a consequence of premacular membrane thickening with accompanying traction on the macula [24]. Operative techniques tailored to individual cases are necessary to achieve anatomical success. However, visual improvement was found to be associated primarily with preoperative visual acuity and the level of subretinal fluid (SRF), not the success of hole closure.

Other vitreomacular interface abnormalities, such as ERM, VMT, or lamellar macular holes (LMH), may be seen in PDR and are likely to progress to MH or macular hole retinal detachment (MHRD), which require surgery [25, 26]. Surgery may obtain a good anatomical outcome, but functional results appear less favorable.

### Neovascular glaucoma (NVG)

The retinal ischemia in PDR patients may lead to neovascularization in the anterior segment and result in NVG. Although PRP and anterior retinal cryotherapy (ARC) may destroy the ischemic retina, early vitrectomy with anti-VEGF and anti-glaucomatous regimens is recommended to prevent irreversible visual loss [16]. In this regard, we propose considering neovascularization of the iris in patients with VH to be an indication for vitrectomy, since early operation, especially when combined with comprehensive PRP, could prevent the development of NVG.

## The surgical principles and tips of diabetic vitrectomy

### The surgical principles of diabetic vitrectomy

**Fig. 2:**
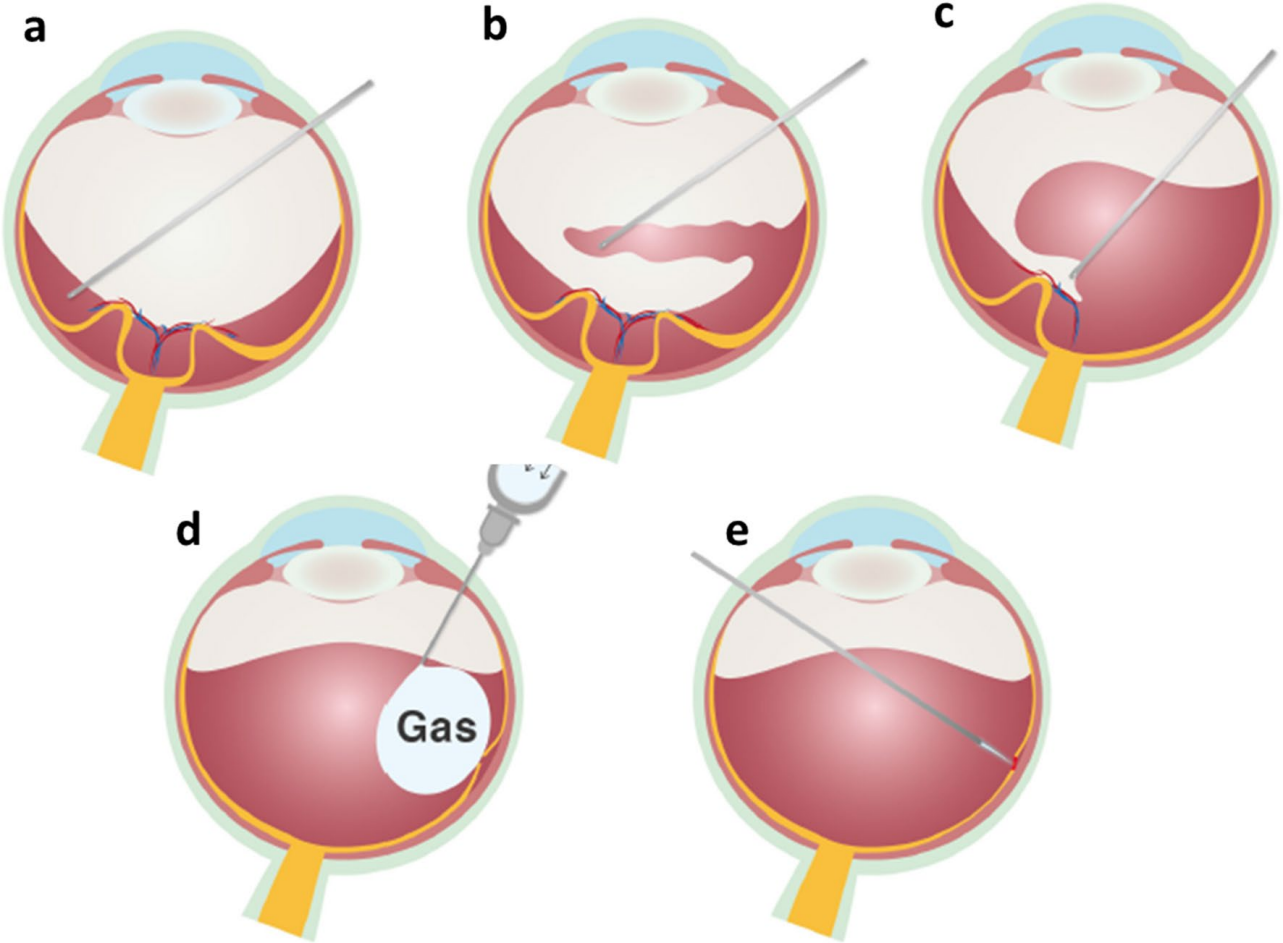
Procedural outline of vitrectomy. **a** After core vitrectomy, the extent of retinal detachment is verified. **b** Careful dissection at a suitable plane is performed to release the anterior-posterior traction force. **c** Tangential traction is further released by removing fibrous tissues. **d** After careful examination of unattended retinal breaks with diathermy, fluid-gas exchange is performed to facilitate retina flattening. **e** Once the vitreous cavity is filled with gas, supplementary photocoagulation is applied to enhance break repair, followed by checking sclerotomy leaks at the end

In a standard three-port vitrectomy (Fig. 2), a core vitrectomy is first performed to gain media clarity, followed by the circumferential release of anterior-posterior traction as necessary and can be safely done. The free edges created here should also facilitate better membrane engagement at different angles during FVP removal, which is performed after clearing subhyaloid hemorrhage. Commonly employed techniques for FVP removal include segmentation, delamination, en bloc dissection, and various bimanual approaches, all of which could be used in combination [10, 16]. In cases of adherent posterior hyaloid attached outside the macula, the practice is to begin tissue dissection in the posterior pole and gradually work up to the periphery as far as can be safely done. When performed carefully with WAVS and MIVS, this traditional “inside-out” maneuver may be substituted with an “outside-in” approach moving from the periphery toward the disc, without jeopardizing the retinal tissue integrity [16].

Bleeding should be checked promptly and rigorously. Hemostasis may be achieved by temporal elevation of the intraocular pressure (IOP), mechanical compression of the bleeding site using a cutter tip or a soft-tipped cannula, endodiathermy, green light endophotocoagulation, or a combination of these. Once all tangential tractional force is released, residual bleeding should be carefully checked by lowering IOP at a gradient to 15 mmHg, which simultaneously examines potential spots of immediate postoperative bleeding. When indicated, ILM peeling in the macular area should follow [20–22]. In TRD, while SRF drainage may not be necessary [27], drainage may achieve earlier macular reattachment for TRD extending beyond the arcades [23] and facilitate retinal reattachment at a 95% rate in high myopia with MH [28], suggesting further study on the applicability for PDR patients. Laser photocoagulation around retinal breaks and PRP (or supplementary PRP) up to the ora serrata is then performed. After appropriate tamponade, sclerotomy sites are checked at the end of surgery and sutured if wound leakage is noted.

### Membrane removal

**Fig. 3:**
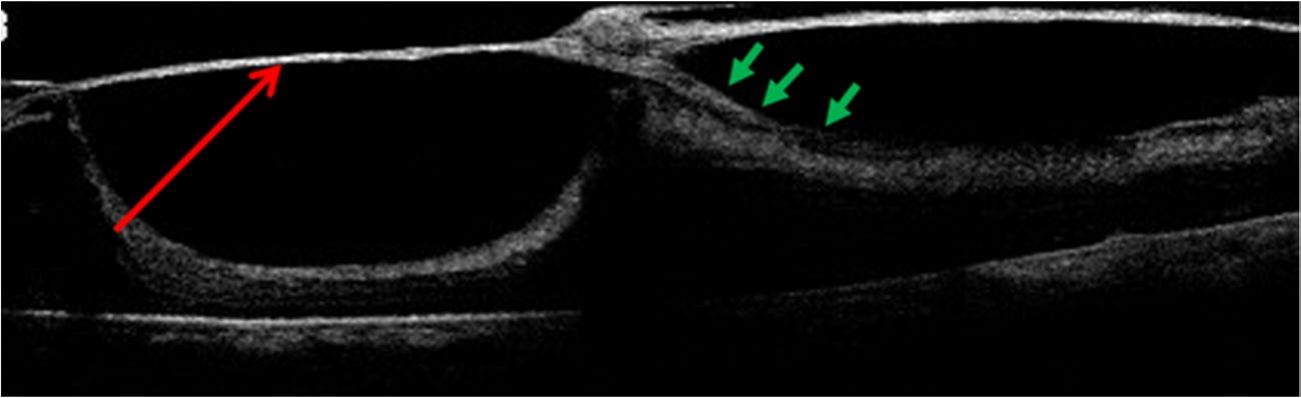
An optical coherence tomography image of ERM and fibrovascular proliferative tissue over the detached retina. The apparent but false surgical plane is indicated by the red arrow, whereas the true surgical plane is indicated by the green arrows

The correct identification of the dissection plane is essential, and to avoid iatrogenic retinal breaks and intraoperative hemorrhage, a gentle movement near the epicenter is necessary. If an adherent thin ERM grows adjacent to and in connection with FVP, it must be lifted to expose the true surgical plane (Fig. 3). Similarly, vitreoschisis and the presence of an underlying membrane should be carefully assessed [16].

Although proliferative tissue should be removed thoroughly to ensure retinal reattachment, this should be balanced with the potential risk of iatrogenic retinal breaks. Therefore, it is sometimes beneficial to leave residual tissues, especially those with tight adhesion along the retinal vessels, when they do not (1) connect to the posterior pole, (2) associate with peripheral retinal breaks, or (3) cause anterior-posterior traction. For complex and broad tissue adhesion not meeting these criteria, retinectomy should be performed when a thorough removal is deemed necessary [18, 29].

In the complex situations of broad adhesion, especially when the membranes are extremely adherent, bimanual dissection is employed. This technique is also likely required in eyes with either iatrogenic or pre-existing retinal breaks with severe proliferation, as the bullous and mobile feature of the retinal detachment may hamper safe, unimanual removal.

### Retinal ablation

Supplementary PRP is commonly applied in PDR patients, especially in CTRRD, up to the posterior of the ora serrata to reduce the risk of fibrovascular ingrowth and delayed postoperative recurrent hemorrhage [6, 17]. A curved probe should facilitate access to less accessible areas such as the anterior superior retina or peripheral retina. The use of an illuminated endolaser, instead of chandelier lighting, and scleral depression may also help peripheral retinal ablation. Currently, cryotherapy for retinopexy is rarely used.

Because of fluid viscosity and retinal rigidity from prolonged traction, residual SRF may localize at sites of retinal detachment and persist after complete membrane removal and air-fluid exchange. Perfluorocarbon liquid may be used to disperse the residual SRF, in order to effectively apply endolaser around retinal breaks located posteriorly. Heavy laser should be avoided to eliminate excessive inflammation and the subsequent complication of extensive chorio-retinal atrophy.

## Specific considerations

### Adjunctive use of anti-VEGF, dexamethasone, and triamcinolone acetonide

Preoperative anti-VEGF is commonly used in patients with active FVP of moderate to severe grades to prevent intraoperative hemorrhage [30]. This single injection could be carried out within a week of the scheduled surgery, although a shorter window of 2–3 days before the surgery is preferred with PDR [18, 29, 31]. Nevertheless, if a high proportion of inactive fibrous proliferation is observed in the tissue, such as those in macular TRS, preoperative anti-VEFG may not be necessary and may even be contraindicated to avoid the induction of fibrovascular regression accompanying fibrous contraction [23, 30].

An intravitreal dexamethasone implant also shows some efficacy for the regression and consolidation of retinal neovascularization, as well as the inhibition of inflammation, thus reducing the formation of postoperative fibrin and ERM [32]. When compared to anti-VEGF, sustained-release steroid shows a reduced risk of tractional responses and is known to remain effective for at least 4 months, thus lasting postoperatively. The advantage, however, should be balanced against the possible increase of IOP after surgery. It is important to note that the implant could become mobile and is preferably placed in the inferior vitreous base [4].

Anti-VEGF or triamcinolone acetonide injection at the end of the surgical procedure was also suggested to lower the risk of subsequent VH, despite concerns of possible delay in vessel repair and, hence, more recurrent hemorrhage [33, 34].

### Non-clearing VH

PRP or ARC has been shown to synergize with anti-VEGF on clearing VH, although broad vitreomacular adhesion is a contraindication for such treatments, especially with ARC [35, 36]. Such eyes are likely to suffer from enhanced proliferative development underneath.

Vitrectomy is much more challenging in eyes with dense VH but without PVD. These eyes may have extensive vitreoretinal adhesions obscuring the dense VH. Careful preoperative evaluation of B-scan is essential to identifying the area of limited PVD for initiating an opening in the posterior hyaloid face. For eyes without any PVD, it is often safer to induce PVD around the disc after core vitrectomy, as the retina there is thicker and less likely to create iatrogenic breaks secondary to PVD induction. Once a PVD is induced, sequential vitrectomy may proceed along the posterior hyaloid surface with the lift and shave technique in avoidance of iatrogenic retinal breaks. Often, the fibrovascular tissue needs to be segmented into several islands before delamination. Our experience has shown that tissue plasminogen activator, or alternatively anti-VEGF, administered 1 week prior to surgery may help the induction of PVD without the risk of excessive bleeding. However, this practice has not been adopted by all surgeons.

### CTRRD

Improved imaging techniques and physician awareness promote better identification of retinal breaks in CTRRD before surgery [29]. Most retinal breaks observed in CTRRD are slit, round, or oval, likely caused by tangential traction force from extensive preretinal FVP. PRP and injections of anti-VEGF, causing a higher percentage of the active fibrovascular component visible in the proliferative tissue, as opposed to the fibrotic dominance reported in our earlier study [37], may play a role in break formation. Also, in our experience, observations of a convex shape in a mobile, detached retina and/or a large detached area disproportionate to the fibrovascular tissue may suggest CTRRD with less prominent breaks or with breaks hidden beneath the membrane. Despite these challenges, vitrectomy performed with MIVS showed a success rate of at least 90% of final retinal reattachment [29, 38]. For complicated proliferation, the bimanual technique may be preferred. Poorer preoperative visual acuity, intraoperative retinectomy, and higher grading of FVP were associated with poorer postoperative visual improvement.

### DME with massive hard exudates

DME with massive hard exudates remains a major challenge in preventing irreversible vision loss, largely due to the subretinal fibrotic plaque from long-standing hard exudate deposition. While retinotomy to remove intraretinal hard exudates could achieve encouraging results [39], a recent study comparing vitrectomy using ILM peeling to nonsurgical treatments observed a marked hard exudates resolution in the vitrectomized group, together with steadily reduced central retinal thickness, fewer anti-VEGF injections, and better visual improvement [40]. A 3-month window for pharmacological treatment is suggested before switching to vitrectomy.

### Macular holes

The strong, multi-directional traction by the ERM, posterior hyaloid, and/or fibrovascular growth around the fovea could induce inner retinal avulsion, LMH, and foveal thinning either from the attached retina or after macular detachment, which may lead to subsequent MH, and in eyes with severe PDR, MHRD [25]. Spontaneous closure of full-thickness MH in PDR might be observed. The location and direction of FVP tractions are more influential than the activity, severity, and extent of FVP in PDR eyes destined to develop full-thickness MH.

Simple ERM and ILM peeling may be used for small to moderate MH with or without shallow RD; the inverted ILM flap technique may be the treatment of choice for other more complex configurations, especially MHRD. In practice, many have resorted to inverted ILM insertion or free ILM flap insertion to achieve hole closure and retinal reattachment [41].

### Retinectomy

**Fig. 4:**
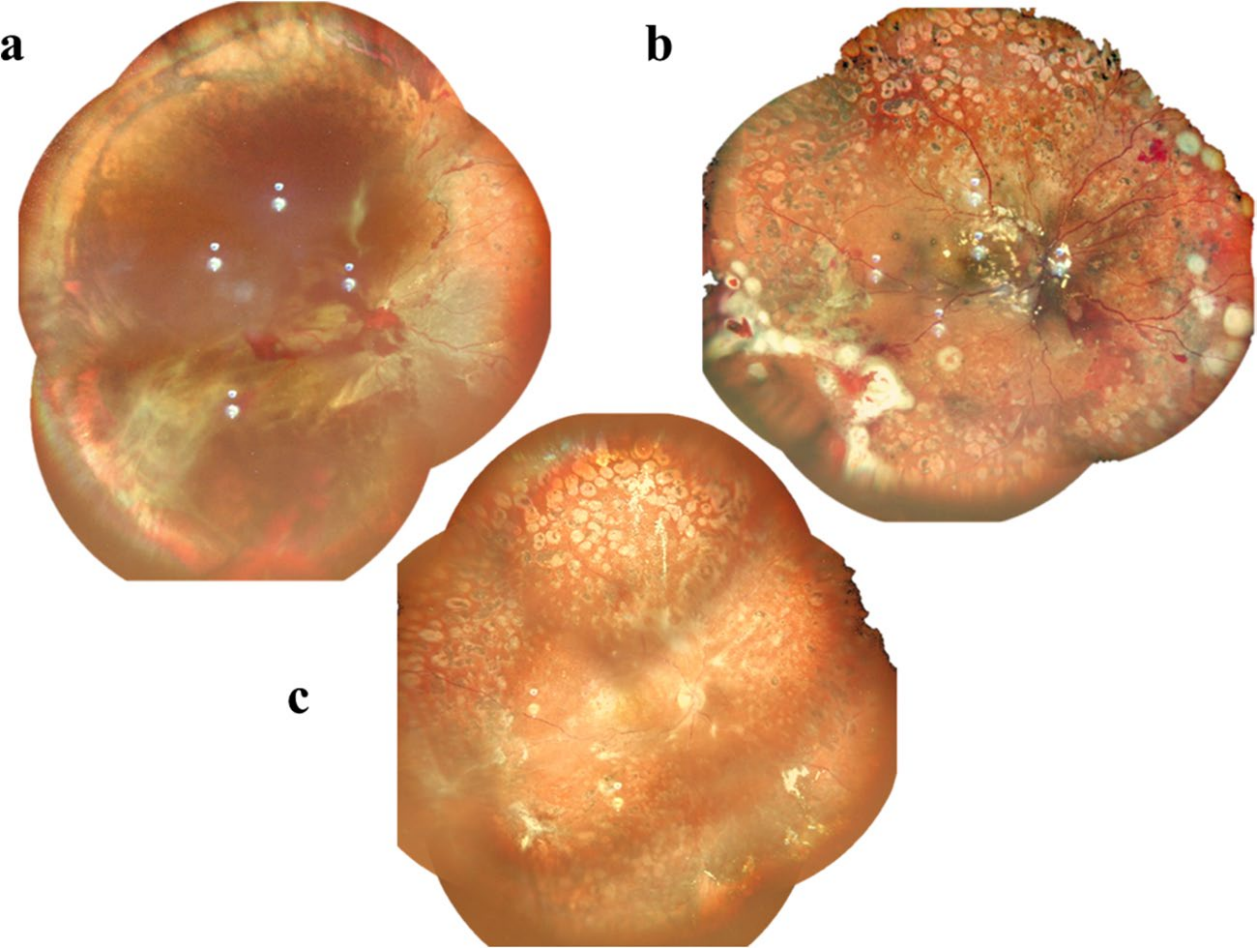
Fundus images of eyes before (**a** and **b)** and after (**c**) peripheral retinectomy

Retinectomy is rarely performed in primary diabetic vitrectomy. However, both peripheral circumferential retinectomy (Fig. 4) and localized midperipheral retinectomy may sometimes be required for traction release and retinal reattachment. The removal of FVP containing complex broad adhesion with multiple epicenters may inevitably create multiple breaks, and a local or limited retinectomy is thus preferred [18, 37, 42]. In some cases, FVP may extend all the way to the far periphery, and scleral buckling may be insufficient to relieve traction and/or reproliferation. Therefore, circumferential retinectomy to remove FVP-induced traction becomes necessary for anatomical success. Silicone oil (SO) infusion is usually needed after these procedures.

### Subretinal bands

**Fig. 5:**
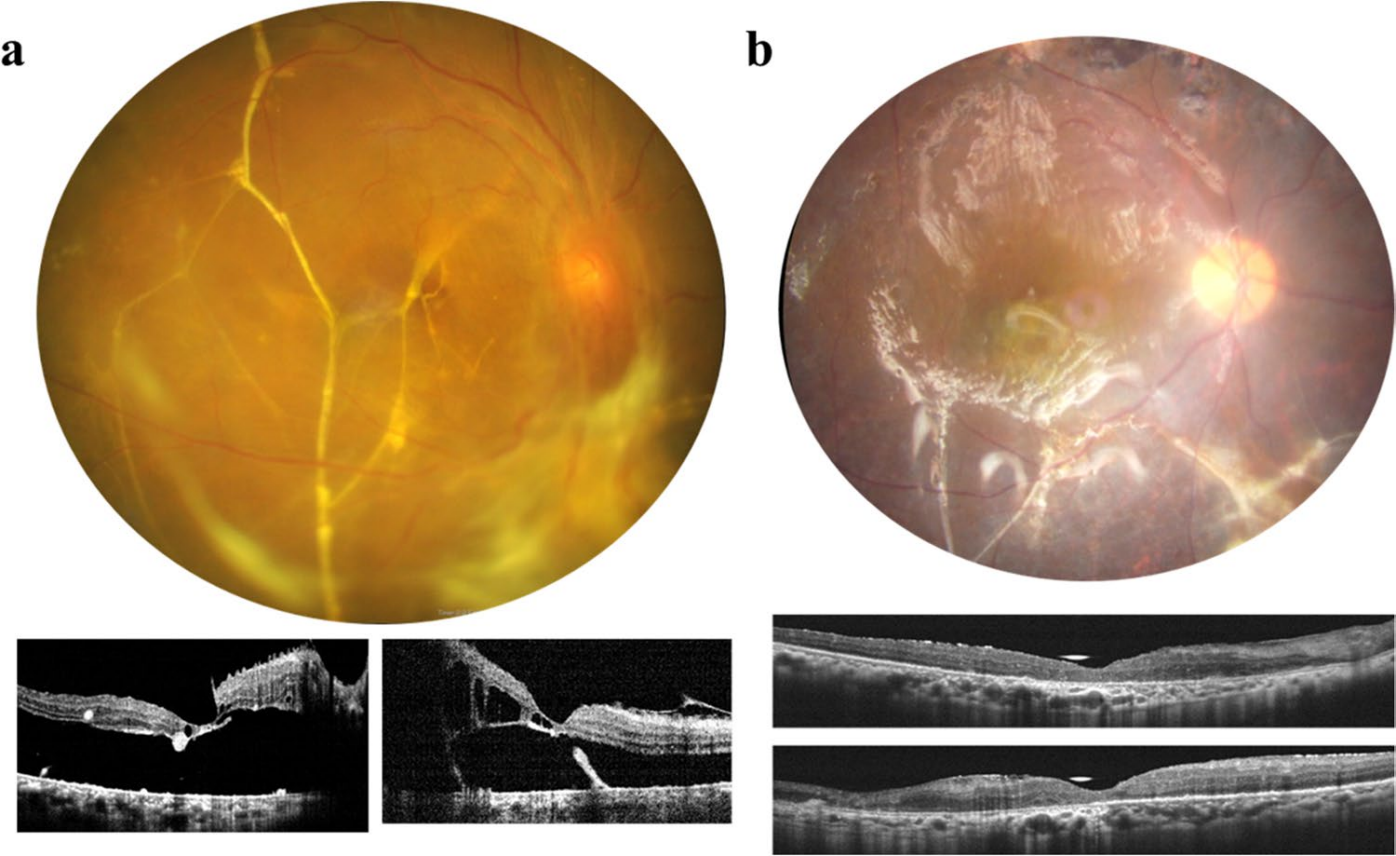
Fundus and optical coherence tomography images of **a** a subretinal band across the macula, and **b** the retina reattached under silicone oil after subretinal band removal

In PDR with retinal detachment, subretinal fibrosis and subretinal bands may develop sooner than those in uncomplicated rhegmatogenous retinal detachment, because of the strong pro-proliferative microenvironment [43]. Subretinal bands located outside the posterior pole may be left undisturbed. However, if proper retinal reattachment is prevented by a band spanning the macula (Fig. 5) or by multiple bands in the mid-periphery, they should be removed or, at the very least, disrupted by microforceps after retinotomy at suitable sites. Sometimes more than one retinotomy is needed for proper traction release.

### SO tamponade in primary vitrectomy

While long-acting gas has been reported as an effective tamponade, it is nevertheless necessary to consider SO as an alternative in extremely complicated cases, such as when there are large retinal breaks, multiple breaks in different quadrants, severe proliferation, extensive retinectomy, or residual vitreoretinal traction. Redetachment rate in these situations was found to be high without long-term SO tamponade [17, 18, 29].

**Fig. 6:**
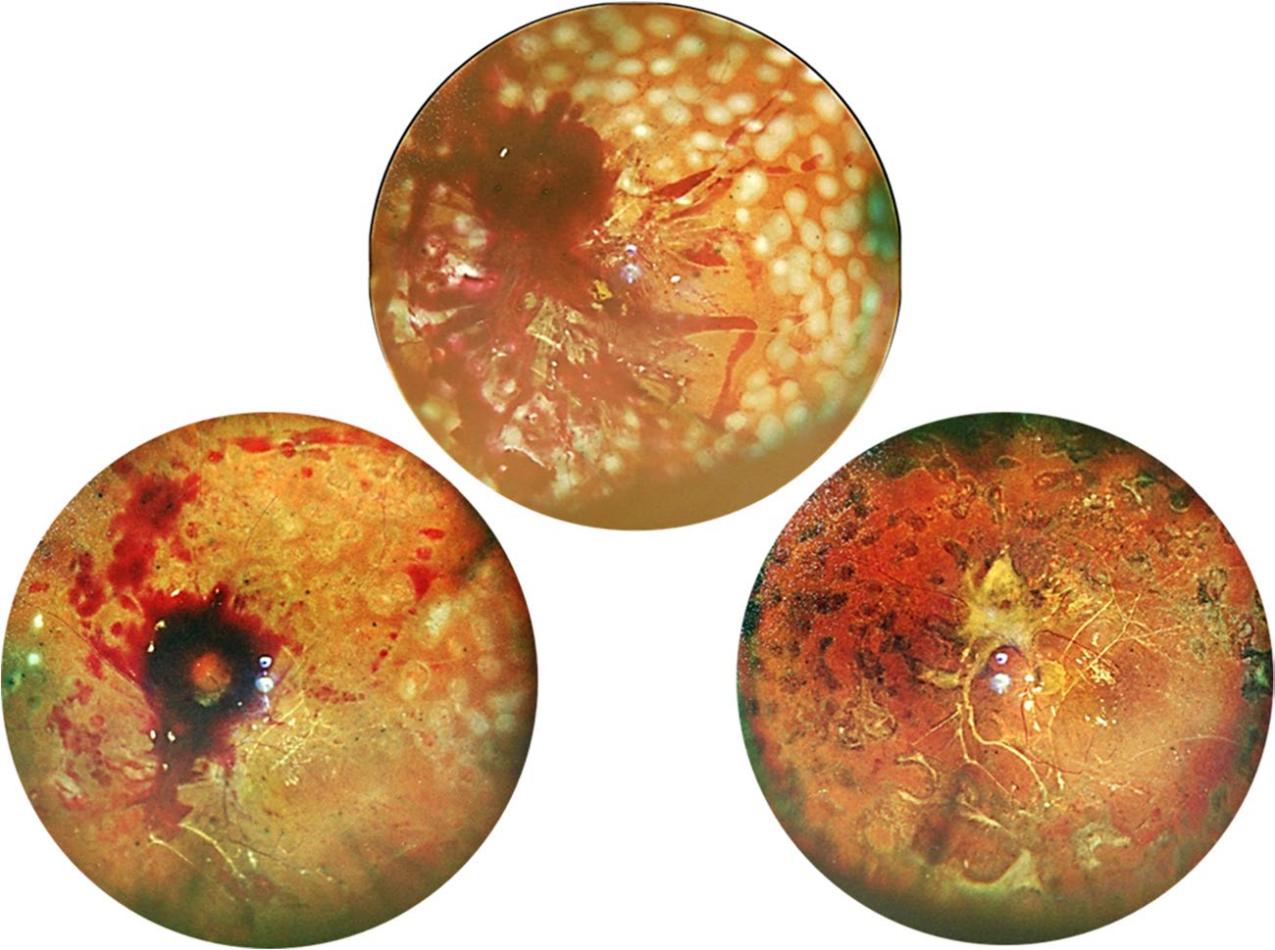
Representative images of thick and localized preretinal hemorrhage under silicone oil which evolved to fibrosis. The thin layered blood is shown in bright red and the thick blood in dark red

All bleeding should be stopped before SO infusion, since clotted blood is likely to promote epiretinal fibrosis and exacerbate macular-threatening traction [29]. In some cases, localized thick fibrosis may develop from a residual blood clot and cause macular traction (Fig. 6), while widespread thick blood from repeated hemorrhage may lead to redetachment, necessitating early intervention, preferably within 1 week, to remove the thick blood and circumvent the inevitable damage to the retina during adherent clot removal. SO may be removed when operation is needed for other indications, such as cataract, macular hole, or macular pucker. We prefer to leave SO for at least 9 months, since delayed removal showed reduced intraocular inflammation and rubeosis [44]. It was also found to be safe with a low rate of retinal redetachment.

### Combined cataract surgery

Combined cataract surgery with vitrectomy may facilitate intraoperative visualization in patients with lens opacification and is popular for faster vision recovery in one single operation. However, it is generally not recommended for fear of excessive postoperative inflammation and stimulating neovascular complications, unless a cataract surgery soon after vitrectomy is anticipated or the lens opacity compromises visualization during vitrectomy [45]. In our experience, the combined procedure should be avoided in eyes with severe retinal ischemia and rubeosis iridis. The scope of the combined procedure and its highly variable prognosis should be thoroughly discussed with the patient.

When a combined cataract removal is considered, complete PRP up to the ora serrata is mandatory, and intravitreal anti-VEGF is highly recommended at the end of operation to prevent postoperative rubeosis. When gas or SO tamponade is used, a prone position may increase the concentration of cytokines and growth factors in the anterior chamber, promoting posterior synechia. In such cases, we routinely perform peripheral iridectomy during surgery to prevent angle closure glaucoma. A patent peripheral laser iridectomy may be difficult to obtain and maintain, even with repeated applications. After surgery, anti-VEGF and/or steroid should be administered as necessary to reduce inflammation, posterior synechia, and neovascularization.

## Postoperative complications

### Recurrent VH

Prolonged postoperative hemorrhage warrants careful examination to rule out rubeosis, RD, NVG, and AHFVP. Uncomplicated cases may be managed by fluid-air exchange, vitreous lavage, and possibly revitrectomy [3, 16]. Intravitreal anti-VEGF and/or particulate steroid injection may facilitate VH reabsorption. Multiple injections may sometimes be required, but the effect of neovascular regression may not be sustained [19]. These procedures may be combined with anterior retinal and sclerotomy site cryotherapy, showing less than 5% recurrence in one study, since neovascularization in these areas may be the main source of rebleeding [46].

### Recurrent RD

Secondary RD is a serious complication rendered inoperable in the presence of multiple large retinal breaks, retinal shortening, opacified cornea, no light perception, and severe NVG. With an overall success rate of over 70%, those operable cases fall into two major categories: pure rhegmatogenous and proliferative tissue-related. The former comes mainly from iatrogenic or postoperative retinal breaks and develops rapidly after primary vitrectomy; the latter develops much later, 40% of which occurring 6 months after surgery, and can be further divided into pure TRD and CTRRD subgroups [47]. In both scenarios, anterior pathology is widely present and mostly requires peripheral retinectomy. Of note, more recurrent detachments have been observed in younger diabetic patients [18, 48]. In the revitrectomy of the proliferative cases, silicone oil tamponade is a better choice than long-acting gas when applied after complete hemostasis [31, 49].

### Macular abnormalities

ERM formation after vitrectomy, observed in over 50% of the cases, has been shown to cause various macular abnormalities in PDR patients, which could be prevented by ERM and ILM peeling in primary vitrectomy [50, 51]. More advanced techniques that combine ERM removal with ILM peeling, ILM flap, inverted flap insertion, and so on are needed to tailor treatment to individual cases [52, 53].

### Central retinal vessel occlusion

As the vitrectomized eyes in PDR patients may not exhibit distinct cherry-red spots and arterial emboli commonly seen in acute central retinal artery occlusion (CRAO), the more reliable indicator for CRAO in these patients could be an increased optical reflectivity from the inner retina in optical coherence tomography and delayed filling time in fluorescein angiography [54]. Considering the difficulties to recognize the early signs of CRAO through ophthalmoscopic means, monitoring high-risk patients closely is essential for timely intervention.

### NVG and elevation of IOP

NVG remains one of the major postoperative complications observed in younger diabetic patients [18, 48], indicating poor surgical prognosis associated with more extensive retinopathy. However, there is a wide array of causes for postoperative IOP elevation, including steroid, inflammation, pupillary block, and unspecified causes. Hence, treatment should be tailored accordingly.

## Conclusion

As diabetes continues to impact public health worldwide, diabetic retinopathy is likely to affect a growing population despite a lowering incidence. Equipped with advanced microincision instruments and powerful imaging systems, vitreoretinal specialists may now perform more delicate maneuvers in PPV for a wider spectrum of PDR. Expanding our understanding of PDR, especially the management of complications such as diabetic maculopathy, cataract-associated conditions, CRAO, and NVG, also helps the timely call for surgeons to perform vitrectomy and increase anatomical and functional success for long-term stabilization. With the findings and new perspectives discussed in the present article, we believe that PPV would serve more PDR patients as an important treatment option for a brighter outlook.
